# TriQuery: A Query-Based Model for Surgical Triplet Recognition

**DOI:** 10.3390/s25175306

**Published:** 2025-08-26

**Authors:** Mengrui Yao, Wenjie Zhang, Lin Wang, Zhongwei Zhao, Xiao Jia

**Affiliations:** 1 School of Control Science and Engineering, Shandong University, Jinan 250100, China; akiliyao7@gmail.com (M.Y.); jiaxiao@sdu.edu.cn (X.J.); 2 Department of Urology, Qilu Hospital of Shandong University, Shandong University, Jinan 250100, China; 3 Cheeloo College of Medicine, Shandong University, Jinan 250100, China

**Keywords:** surgical triplet recognition, query-based learning, transformer, surgical video analysis, multi-task learning

## Abstract

Artificial intelligence has shown great promise in advancing intelligent surgical systems. Among its applications, surgical video action recognition plays a critical role in enabling accurate intraoperative understanding and decision support. However, the task remains challenging due to the temporal continuity of surgical scenes and the long-tailed, semantically entangled distribution of action triplets composed of instruments, verbs, and targets. To address these issues, we propose TriQuery, a query-based model for surgical triplet recognition and classification. Built on a multi-task Transformer framework, TriQuery decomposes the complex triplet task into three semantically aligned subtasks using task-specific query tokens, which are processed through specialized attention mechanisms. We introduce a Multi-Query Decoding Head (MQ-DH) to jointly model structured subtasks and a Top-K Guided Query Update (TKQ) module to incorporate inter-frame temporal cues. Experiments on the CholecT45 dataset demonstrate that TriQuery achieves improved overall performance over existing baselines across multiple classification tasks. Attention visualizations further show that task queries consistently attend to semantically relevant spatial regions, enhancing model interpretability. These results highlight the effectiveness of TriQuery for advancing surgical video understanding in clinical environments.

## 1. Introduction

Minimally invasive surgeries, such as laparoscopy and arthroscopy, are increasingly recorded using high-resolution intraoperative video systems. These videos contain rich spatiotemporal information that, if accurately and efficiently parsed, can support intraoperative decision-making, postoperative skill evaluation, and complication risk prediction [1]. In robotic surgeries such as those conducted with the da Vinci system, such analysis can further enhance manipulation precision and safety. Ultimately, the development of autonomous surgical systems hinges on reliable video-based scene understanding.

Among various video understanding tasks, surgical triplet recognition has gained increasing attention for its ability to interpret fine-grained surgical interactions by identifying structured triplets in the form of instrument–verb–target (e.g., hook–dissect–gallbladder). This structured information supports real-time risk alerts, objective skill assessment, and prior knowledge and safety constraints for intelligent surgical systems [2,3].

However, surgical triplet recognition in video analysis remains challenging due to two main factors. The first is the class imbalance in triplet distributions, where a few frequent combinations dominate and many rare but clinically important ones are underrepresented. The second is the temporal inconsistency caused by factors like bleeding, tool switching, and abrupt camera motion, which disrupt the coherence of frame-level predictions. Moreover, clinical deployment demands model interpretability, necessitating visual explanations such as attention maps or saliency regions.

In recent years, deep learning has significantly advanced surgical video understanding. Convolutional Neural Network (CNN) and Recurrent Neural Network (RNN) architectures have been widely applied but often struggle with capturing both fine spatial details and long-range temporal dependencies [4,5]. Transformer-based models, especially the Swin Transformer and its lightweight variant Swin Transformer Tiny (Swin-T), offer improved multi-scale context modeling through hierarchical attention mechanisms [6,7,8]. Multi-task learning and temporal attention methods have also been explored to better represent complex surgical interactions [9,10,11,12]. Despite these advances, current methods often overlook the dual challenges of long-tail distributions and temporal instability. Many operate at the frame level and lack mechanisms for semantic disentanglement and robust temporal modeling, limiting their generalizability and reliability in real-world clinical settings. A more comprehensive review of related methods is provided in Section 2 to highlight their strengths and limitations in detail.

In this paper, we propose TriQuery, a lightweight and query-based framework tailored for fine-grained surgical triplet recognition. TriQuery integrates several carefully designed components to handle both spatial and temporal challenges in surgical video analysis. Specifically, it leverages a Swin-T-based backbone for multi-scale visual encoding, enabling effective extraction of spatial and contextual features from high-resolution surgical frames. To handle the problem of class imbalance and semantic entanglement, we design a Multi-Query Decoding Head (MQ-DH) that introduces structured multi-task learning. By associating task-specific query groups with instruments, verbs, and targets, MQ-DH enables semantic disentanglement and strengthens the model’s ability to generalize across underrepresented categories. To address temporal inconsistency, we propose the Top-K Guided Query Update (TKQ) module, which selectively reuses high-confidence queries from the previous frame in a lightweight manner. This design enhances temporal coherence across frames without incurring significant computational overhead, ensuring stable and efficient inference. Furthermore, TriQuery supports interpretable attention visualization by exposing cross-attention weights between queries and image features, offering insights into the model’s decision process and supporting trustworthy deployment in clinical settings.

Our contributions are summarized as follows:

1. We propose TriQuery, a query-centric multi-task framework for surgical video recognition. It jointly models instruments, verbs, targets, and their triplet combinations through task-specific queries and a dedicated MQ-DH module, enabling semantic disentanglement and mitigating long-tailed class imbalance.
2. We introduce the TKQ module, which reuses high-confidence queries from the previous frame to guide current decoding, enhancing temporal consistency with minimal overhead.
3. Our framework achieves superior performance on the CholecT45 dataset, outperforming baseline methods while providing attention visualizations that facilitate clinical applicability.

## 2. Related Work

Building on the challenges and motivations outlined in Section 1, this section reviews the relevant prior work in surgical video analysis and related domains. We focus on four methodological directions: spatiotemporal modeling strategies, windowed vision transformers, learnable query-based structured recognition, and temporal consistency modeling.

**Spatiotemporal Modeling with Convolutional and Recurrent Networks.** Early approaches typically decomposed videos into individual frames, employing 2D CNNs for spatial feature extraction and using RNNs or temporal convolutions for sequential modeling. Alternatively, 3D CNN-based models such as C3D and I3D were introduced to jointly learn spatiotemporal representations. However, these methods struggle to capture long-range semantic dependencies and often fail to model multi-scale features effectively. Moreover, their computational complexity tends to increase significantly with longer temporal windows. To address these limitations, recent studies have explored enhancing CNN backbones with attention modules to improve feature expressiveness [13,14]. Wu et al. [15] proposed a CNN–self-attention hybrid architecture for wireless signal recognition, demonstrating that attention mechanisms can boost discriminative capacity even in non-visual domains. These findings further support the incorporation of self-attention into spatial feature extractors for video-based recognition tasks.

**Windowed Vision Transformers.** Swin Transformer [6] introduces a window-based self-attention scheme that computes attention within local non-overlapping windows. Through shifted windowing across layers, it enables global information exchange while maintaining computational scalability. This architecture has demonstrated strong performance in medical image segmentation and detection tasks. However, its single-scale token representation may struggle to capture small structures or subtle semantic cues, especially in scenarios with class-imbalanced distributions. In related visual modeling work, Xu and Wang [16] proposed a cascaded non-local mean network with dual-path fusion for single-image super-resolution, underscoring the value of multi-branch architectures and non-local interactions. Their findings emphasize the importance of complementary pathways and long-range dependencies in preserving fine-grained information, which aligns with the challenges faced in surgical action recognition under low-resolution or cluttered visual conditions.

**Learnable query-based structured recognition.** Inspired by the DEtection TRansformer (DETR) [17], learnable query-based paradigms have been adapted for structured recognition tasks across domains. In surgical video analysis, Nwoye et al. [18] proposed Rendezvous, a query-based model that adopts separate sets of task-specific queries to independently recognize instruments, verbs, and targets, enabling explicit modeling of role-specific semantics. While this design offers strong modularity and interpretability, it lacks mechanisms for inter-frame temporal modeling and operates on single-scale spatial features. As a result, it struggles with temporal discontinuities and ambiguous instrument–verb–target associations under motion blur or occlusion. Similarly, Tufail et al. [19] leveraged task-specific queries for document layout and entity extraction, and Liu et al. [20] employed query-driven target generation in video keyframes. These efforts underscore the versatility of query mechanisms across modalities. However, most existing approaches process frames independently and overlook temporal dynamics, limiting their robustness in visually complex or temporally unstable scenarios.

**Temporal Consistency and Inter-frame Modeling.** To enhance temporal reasoning in video understanding, prior works have explored approaches such as optical flow alignment, two-stream architectures, and temporal Transformers [21,22]. While these methods effectively capture motion dynamics, they often incur high computational costs, are sensitive to occlusions, and are less suited for clinical deployment. Moreover, most existing query-based models treat frames independently and lack mechanisms for lightweight temporal integration within the query space, limiting their ability to enforce temporal continuity or smooth predictions across frames.

## 3. Methods

In this paper, we propose TriQuery, a lightweight and query-based framework for surgical video action recognition. It is designed to address the challenges of class imbalance with semantic entanglement and temporal inconsistency in surgical video analysis by integrating a Swin-T visual encoder, a multi-query attention decoder, and a cross-frame guidance module. TriQuery takes each laparoscopic video frame as input and performs multi-task classification to predict the instrument–verb–target triplet and its individual components.

### 3.1. TriQuery Architecture Overview

**Figure 1:**
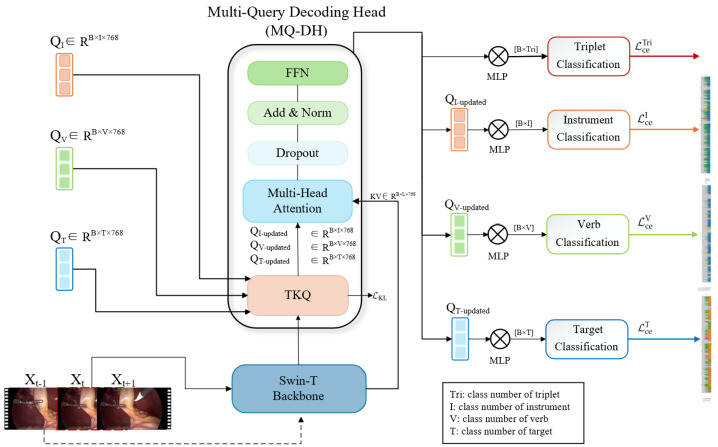
Overview of the proposed TriQuery framework. TriQuery features two key modules: a Multi-Query Decoding Head (MQ-DH) for structured multi-task classification (triplet, instrument, verb, target) and a Top-K Guided Query Update (TKQ) module for lightweight temporal consistency. The Swin-T backbone extracts hierarchical features, which are attended by task-specific queries. $X_{t-1},X_{t},X_{t+1}$ denote three consecutive video frames, each processed individually through the Swin-T backbone to extract hierarchical features. TKQ injects temporal priors by reusing top-*K* queries from the previous frame to refine current predictions. FFN and MLP denote feedforward networks and multi-layer perceptrons, respectively.

An overview of the TriQuery architecture is illustrated in Figure 1. TriQuery is an end-to-end framework that integrates three core components: a Swin-T backbone for visual encoding, a Multi-Query Decoding Head (MQ-DH) module for structured multi-task classification, and a Top-K Query Update (TKQ) module for lightweight temporal consistency enhancement.

Given a sequence of laparoscopic video frames as input (e.g., $X_{t-1},X_{t},X_{t+1}$), TriQuery first employs the Swin-T encoder to extract hierarchical multi-scale spatial features. These features are then processed by the MQ-DH module, which supports four parallel classification tasks: one triplet classifier and three auxiliary branches for the recognition of instrument, verb, and target. The three auxiliary branches are equipped with dedicated sets of learnable task-specific queries, which interact with the shared visual features through cross-attention mechanisms, enabling the model to distill semantically disentangled representations for each subtask. The triplet classification head operates without explicit query interaction but benefits from the supervision signals and feature regularization provided by the auxiliary branches. To enhance temporal stability, the TKQ module uses the top-K high-confidence query predictions from the previous frame and fuses them with the current frame’s queries through residual addition. This mechanism preserves causal inference while injecting temporal priors to smooth frame-wise predictions. The entire model is jointly optimized using a combination of cross-entropy loss for classification and Kullback–Leibler (KL) divergence loss between consecutive predictions to enforce temporal consistency and reduce prediction jitter. Further details are provided in the following sections.

### 3.2. Swin-T Backbone for Hierarchical Visual Feature Extraction

We adopt Swin-T as the backbone of TriQuery for hierarchical visual feature extraction. Each video frame is first partitioned into non-overlapping 4×4 patches, which are flattened and linearly projected into fixed-dimensional embeddings. These embeddings are then processed by a four-stage encoder composed of Swin Transformer blocks. This backbone is also used as a baseline model in our ablation studies to facilitate fair comparisons with TriQuery’s enhanced variants.

Each block alternates between window-based multi-head self-attention (W-MSA) and shifted window multi-head self-attention (SW-MSA). W-MSA operates locally within each window to preserve fine-grained spatial details, while SW-MSA introduces cross-window interactions via a cyclic shift strategy. This design effectively balances local spatial precision with global contextual awareness.

As the encoding proceeds through stages, the model progressively downsamples the feature maps and expands the semantic receptive field, producing multi-scale and multi-level contextual features that are well-suited for downstream query-based decoding and classification tasks. The feature outputs are projected to a common dimensionality and fed into the MQ-DH module, enabling the model to extract fine-grained and high-level semantic information effectively.

### 3.3. Multi-Query Decoding Head for Structured Multi-Task Learning

To address the structured nature of surgical action recognition, particularly the long-tailed distribution and entangled label semantics inherent in triplet annotations, we propose the MQ-DH composed of four classification branches. These include a direct 100-class triplet classifier and three auxiliary query-based branches that separately predict instruments (6 classes), verbs (10 classes), and targets (15 classes). The number of classes in each subtask follows the category definitions provided in the CholecT45 dataset, as described in Section 4.1. The auxiliary branches decompose each action triplet into its semantic components (e.g., instrument, verb, and target) and utilize dedicated groups of learnable task-specific queries. By jointly modeling component-level and triplet-level semantics, the MQ-DH facilitates multi-task learning and helps mitigate the effects of class imbalance.

Each query group interacts with shared visual features extracted from the Swin-T backbone through a Transformer-based cross-attention mechanism, enabling task-oriented semantic representation learning. This design promotes semantic disentanglement across subtasks, allows for direct supervision on individual components, and helps to alleviate the long-tailed distribution problem by decoupling rare triplets into more balanced subtasks, thereby improving generalization on underrepresented classes.

To support multi-task learning in parallel, each input frame $X_{t}$ is first passed through the backbone to produce patch-level visual features $X_{t}\in R^{L\times C}$, where *L* is the number of image patches and *C* the feature dimension. For each task τ∈{triplet,instrument,verb,target}, a set of learnable query vectors $Q^{\left(\tau \right)}\in R^{K_{\tau }\times C}$ is defined, where $K_{\tau }$ denotes the number of classes for task τ. The task-specific cross-attention is computed as follows:

(1) $${\mathrm{Attn}}^{\left(\tau \right)}\left(Q^{\left(\tau \right)},X_{t}\right)=\mathrm{Softmax}\left(\frac{Q^{\left(\tau \right)}W_{Q}{\left(X_{t}W_{K}\right)}^{⊤}}{\sqrt{d}}\right)\left(X_{t}W_{V}\right),$$

where $W_{Q}$, $W_{K}$, and $W_{V}\in R^{C\times d}$ are learnable projection matrices for the query, key, and value embeddings, and *d* is the attention head dimension.

The resulting attention features are refined through residual connections, layer normalization (LN), and a feedforward network (FFN) to produce the task-specific representations:

(2) $${\tilde{Q}}^{\left(\tau \right)}=\mathrm{FFN}\left(\mathrm{LN}\left(Q^{\left(\tau \right)}+{\mathrm{Attn}}^{\left(\tau \right)}\right)\right).$$

Each query group is followed by a lightweight classification head, and all tasks are jointly optimized using a weighted cross-entropy loss, which ensures multi-task consistency and addresses label imbalance. Additionally, the learned attention weights provide interpretability by highlighting task-specific spatial focus regions, enabling the generation of attention maps that offer insights into model behavior and support clinical trust. The output ${\tilde{Q}}^{\left(\tau \right)}$ from each task-specific decoding head is further pooled and flattened to obtain a compact task embedding $f\in R^{C}$, which is then fed into the corresponding classification heads for final prediction.

### 3.4. Top-K Guided Query Update for Temporal Coherence

**Figure 2:**
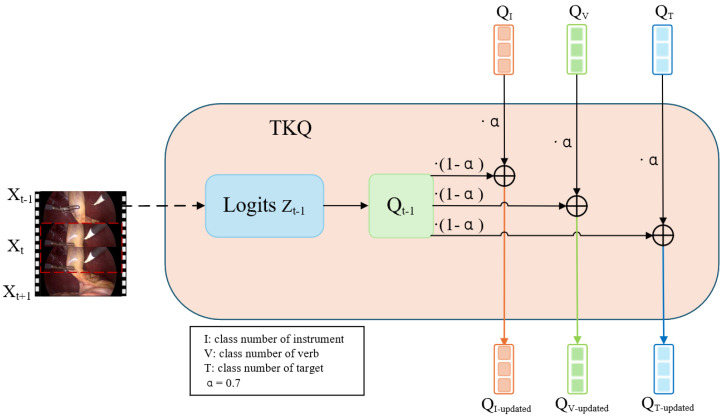
Illustration of the TKQ module. Given the current frame $X_{t}$ and the previous frame $X_{t-1}$, the TKQ module injects temporal priors into task-specific queries. Logits $Z_{t-1}$ from the previous frame are used to identify top-K confident predictions, from which corresponding query embeddings $Q_{t-1}$ are retrieved. These historical queries are blended with the current learnable query embeddings $Q_{I}$, $Q_{V}$, $Q_{T}$ for the instrument, verb, and target branches, respectively. The updated queries $Q_{I-updated}$, $Q_{V-updated}$, $Q_{T-updated}$ are then used for cross-attention in downstream classification heads.

To enhance temporal consistency and preserve semantic continuity across consecutive video frames, we introduce a lightweight TKQ module. During both training and inference, the top-5 class predictions from the previous frame’s triplet task logits are selected. This choice is based on the empirical observation that each frame in the CholecT45 dataset typically contains no more than three distinct action components (i.e., instruments, verbs, or targets). Selecting five top predictions for each component group ($Q_{I}$, $Q_{V}$, $Q_{T}$) allows for a small buffer to capture potentially emerging or uncertain actions while avoiding excessive noise. The corresponding query vectors are then used to guide the initialization of the current frame’s triplet task queries via a residual fusion mechanism. This design enables soft temporal memory propagation across frames, improving frame-level stability without relying on recurrent structures. The detailed flow of the TKQ module is illustrated in Figure 2, which shows how the queries are updated in our model.

Specifically, the previous frame $X_{t-1}$ is passed through the shared backbone and the decoder to obtain the class logits $Z_{t-1}\in R$. The top class indices T with the highest confidence scores are selected, and their corresponding query vectors ${\left\{q_{t-1}^{\left(k\right)}\right\}}_{k\in T}$ from the embedding table are projected to the decoder dimension D=768 via a linear transformation.

These selected vectors are then integrated into the current frame’s query set $Q_{t}$ through residual blending:

(3) $$Q_{t}\left[T\right]\leftarrow \alpha \cdot Q_{t}\left[T\right]+\left(1-\alpha \right)\cdot q_{t-1}^{\left(T\right)},$$

where α is a predefined fusion weight selected empirically, which is set to 0.7 to balance temporal continuity from the previous frame with adaptability to current frame features.

This fusion injects temporal priors into the current queries while preserving their adaptability, thereby promoting smooth transitions across frames. The mechanism introduces a lightweight and causal form of temporal memory without relying on explicit motion modeling (e.g., optical flow). The enhanced queries then attend to current visual features through cross-attention.

To further stabilize temporal dynamics, we impose a KL divergence constraint between consecutive frame predictions, which penalizes abrupt changes and regularizes inter-frame consistency. Compared to heavy temporal models or frame-stacking strategies, the proposed TKQ module adds minimal computational overhead while substantially improving prediction smoothness and robustness, particularly in challenging scenarios involving occlusions, camera motion, or frequent tool switching. Furthermore, the design preserves causality, making it well-suited for online inference settings.

### 3.5. Loss Function

The total loss $L_{\mathrm{total}}$ is designed to jointly optimize all four classification branches unified under the MQ-DH, including the triplet classification and three auxiliary subtasks for instrument, verb, and target recognition. Each branch is supervised using an independent cross-entropy loss, and their contributions are combined as a weighted sum. In addition, we incorporate KL divergence loss between the triplet prediction distributions of consecutive frames to promote temporal consistency across video frames. The final training objective is formulated as

(4) $$L_{\mathrm{total}}=\lambda_{1}L_{\mathrm{ce}}^{\mathrm{Tri}}+\lambda_{2}L_{\mathrm{ce}}^{I}+\lambda_{3}L_{\mathrm{ce}}^{V}+\lambda_{4}L_{\mathrm{ce}}^{T}+\lambda_{5}L_{\mathrm{KL}},$$

where $L_{\mathrm{ce}}^{\mathrm{Tri}}$ is the binary cross-entropy loss for overall multi-label recognition, $L_{\mathrm{ce}}^{I},L_{\mathrm{ce}}^{V},L_{\mathrm{ce}}^{T}$ are the task-specific classification losses, and $L_{\mathrm{KL}}$ is the KL divergence loss between the prediction distributions of the current frame and the previous frame. The weights $\lambda_{1}$ to $\lambda_{5}$ are empirically chosen to balance classification and temporal regularization.

During inference, the model first extracts spatial–temporal features from each input frame using the Swin Transformer backbone. The feature map is processed with global average pooling to produce a compact representation $f\in R^{C}$. This representation is passed to four independent classification heads to produce logits for each task:

(5) $$\begin{array}{cc} {\hat{y}}_{\mathrm{Tri}} & =\sigma (W_{\mathrm{Tri}}\cdot f+b_{\mathrm{Tri}}),\\ {\hat{y}}_{I} & =\sigma (W_{I}\cdot f+b_{I}),\\ {\hat{y}}_{V} & =\sigma (W_{V}\cdot f+b_{V}),\\ {\hat{y}}_{T} & =\sigma (W_{T}\cdot f+b_{T}), \end{array}$$

where σ(·) denotes the element-wise sigmoid function, and W and b are learnable parameters of the linear classifier heads. The final multi-label predictions are obtained by thresholding the outputs:

(6) $$\tilde{y}=I\left(\hat{y}>0.5\right),$$

where I(·) is the indicator function, and the function is used to convert the predicted probability into the encoding of the predicted label.

## 4. Experimental Setup

### 4.1. Dataset

We conduct experiments on the CholecT45 dataset [18], a fine-grained surgical video action recognition benchmark specifically designed for laparoscopic cholecystectomy procedures. CholecT45 is derived from the CholecT50 collection and was officially released in April 2022. It comprises 45 endoscopic videos recorded at 1 FPS, with a total of 90,489 annotated frames and 127,385 labeled triplet instances. Each frame is annotated with one or more action triplets in the form of instrument–verb–target, comprising 100 unique combinations derived from 6 instrument classes, 10 verb classes, and 15 target classes.

Compared to earlier surgical datasets with coarse-grained annotations (e.g., surgical phases), CholecT45 adopts a fine-grained and semantically structured labeling scheme, enabling a more detailed understanding of surgical actions. The dataset also provides mapping files to recover individual component labels, enabling structured learning.

**Figure 3:**
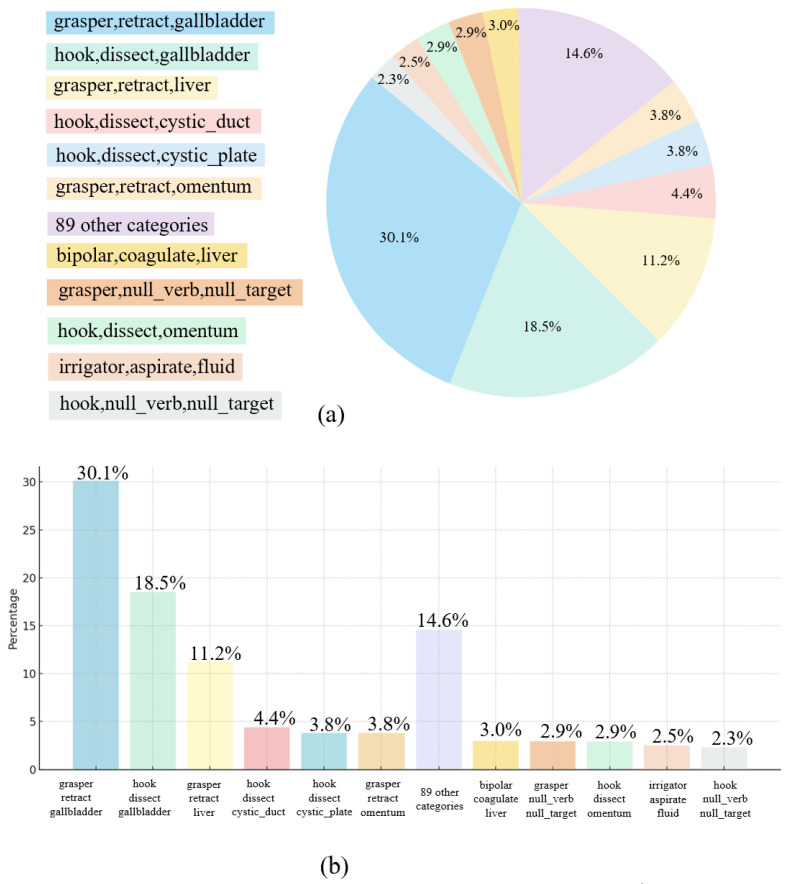
Illustration of imbalanced surgical behavior categories. (**a**) The pie chart presents the proportion of instances for each surgical procedure triplet class. (**b**) The bar chart displays the same classes sorted by frequency, using the same color scheme as the pie chart for visual coherence. The two most frequent triplet classes account for nearly half of all instances, while 89 classes with proportions below 2% collectively contribute only 14.6% of the total, highlighting the severity of the class imbalance.

To highlight the class imbalance in CholecT45, we visualize the distribution of triplet classes using both a pie chart and a bar chart, as shown in Figure 3. The two most frequent classes account for nearly half of all instances, while the 89 least frequent classes together comprise only 14.6% of the dataset. This long-tailed distribution presents difficulties for multi-class classification tasks.

### 4.2. Evaluation Metrics

To ensure fair and reproducible evaluation, we follow the standard five-fold cross-validation protocol recommended for CholecT45 [23]. Each fold contains 36 videos for training and 9 for testing, and we report the averaged results over all five folds. All models are trained and evaluated under this consistent setting.

We evaluate all classification tasks (triplets and subtasks), using the accuracy score (Acc), F1-score (F1), and average precision (AP). For consistency, metric names are reused across tasks without explicit task-specific notation and are assumed to refer to the current task under evaluation unless otherwise stated.

Given the ground-truth label matrix $Y\in {\left\{0,1\right\}}^{N\times C}$ and the predicted binary label matrix $\hat{Y}\in {\left\{0,1\right\}}^{N\times C}$, where *N* is the number of samples and *C* is the number of classes, the accuracy score is defined as

(7) $$\mathrm{Acc}=\frac{1}{N\cdot C}\sum_{i=1}^{N}\sum_{j=1}^{C}I\left(y_{ij}={\hat{y}}_{ij}\right),$$

where I(·) is the indicator function. The F1-score is computed based on the total number of True Positives (TP), False Positives (FP), and False Negatives (FN) over the evaluation set, using the standard formula

(8) $$\begin{array}{cc} F1 & =\frac{2\cdot \mathrm{Precision}\cdot \mathrm{Recall}}{\mathrm{Precision}+\mathrm{Recall}}, \end{array}$$

(9) $$\begin{array}{cc} \mathrm{Precision} & =\frac{TP}{TP+FP+\epsilon }, \end{array}$$

(10) $$\begin{array}{cc} \mathrm{Recall} & =\frac{TP}{TP+FN+\epsilon }, \end{array}$$

with ϵ being a small constant to prevent division by zero. The AP is computed as the area under the precision–recall curve for each class and then averaged across all classes:

(11) $$\mathrm{AP}=\frac{1}{C}\sum_{j=1}^{C}\int_{0}^{1}p_{j}\left(r\right)\;dr,$$

where $p_{j}\left(r\right)$ denotes the precision as a function of recall *r* for class *j*.

### 4.3. Implementation Details

All experiments were conducted on an NVIDIA A100-SXM4-40GB GPU. We employ the AdamW optimizer with $\beta_{1}=0.9$, $\beta_{2}=0.999$, an initial learning rate of $2\times 10^{-4}$, and weight decay of 0.05. A cosine annealing learning rate schedule is used with 2 warmup epochs. The batch size is set to 32, and gradient accumulation is used (2 steps) to simulate a larger batch size while keeping memory consumption manageable. The training is stabilized using gradient clipping with a max norm of 1.0. The model is built upon the Swin-T backbone, which performs hierarchical feature extraction with local window-based multi-head self-attention. Based on this backbone, we further introduce a multi-task attention module to jointly model the instrument, verb, and target in a query-based framework. The dataset is split into five folds for cross-validation, and each fold is trained for 10 epochs. To mitigate overfitting, a dropout rate of 0.3 is applied to the Transformer layers. The loss function used is binary cross-entropy (BCE) with logits, appropriate for multi-label triplet prediction.

## 5. Experimental Results

### 5.1. Comparison with State-of-the-Art Methods

In this experiment, we evaluate the effectiveness of our proposed TriQuery framework on the CholecT45 dataset by conducting comparative experiments against several widely adopted action recognition methods, including CNN+RNN [4], CNN–LSTM (Long Short-Term Memory) [24], Dual-stream CNN [25], 3D CNN [5], Rendezvous [18], and the Swin-T baseline [6]. Detailed results are presented in Table 1 and Table 2.

We present the triplet classification results in Table 1, which compares several state-of-the-art methods on the CholecT45 dataset using five-fold cross-validation. For clarity, results from the first three folds are shown along with the average performance. Our proposed TriQuery model outperforms the competing approaches, achieving an accuracy of 71.53% in the 100-way triplet classification task. Compared to Rendezvous (69.11%), CNN+RNN (69.59%), CNN-LSTM (70.54%), Dual-stream CNN (70.78%), and 3D CNN (70.97%), TriQuery demonstrates superior performance, highlighting the strength of our multi-task Transformer-based architecture in modeling both local and global semantics. Notably, TriQuery improves upon the Swin Transformer baseline (68.10%) by 3.43%, demonstrating the effectiveness of our query-driven decoder and temporal consistency modeling. Furthermore, TriQuery achieves an AP of 45.19%, outperforming both Rendezvous (37.98%) and the Swin baseline (40.15%), reflecting its advantage in handling fine-grained semantic associations.

Table 2 shows the results on the three sub-classification tasks—instrument (6 classes), verb (10 classes), and target (15 classes)—averaged across all five folds. TriQuery consistently achieves high performance across the three subtasks as well as the triplet task, validating the effectiveness of its multi-query decoding mechanism for structured multi-task learning. These results also confirm that the Swin-T-based backbone outperforms conventional CNN architectures and that our query-augmented design brings further gains in fine-grained surgical activity recognition.

### 5.2. Attention Map Visualization

To enhance interpretability and gain deeper insights into the semantic behavior and spatial focus of our model, we visualize the attention distributions produced by the task-specific query vectors in the decoding heads.

**Figure 4:**
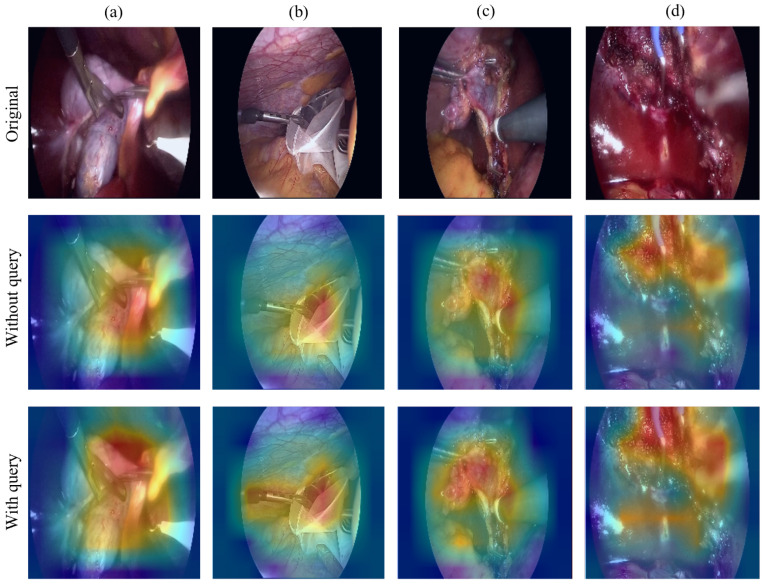
Visualization of attention maps guided by query-based decoding. (**a**–**d**) present four representative surgical frames. The first row shows original images. The second row shows attention maps from the triplet classification branch without incorporating auxiliary queries. The third row presents refined attention maps after integrating the multi-query decoding modules, including task-specific queries for instrument, verb, and target recognition. The enhanced focus on semantically relevant regions demonstrates the effectiveness of our multi-query design.

Figure 4 compares the attention maps from the triplet classification branch without auxiliary queries (second row) and those obtained after integrating the multi-query modules (third row). The first row shows the original surgical video frames, over which attention maps are overlaid to better illustrate the spatial distribution of focus. When auxiliary queries are not used, the attention is relatively diffuse and often fails to concentrate on semantically meaningful regions, which can affect classification accuracy. For example, in the rightmost sample (d), a surgical hook appears on the right side of the original image but receives little attention in the second row. After applying the multi-query modules, the attention map in the third row clearly highlights this area, indicating improved semantic alignment. Moreover, the full model exhibits a stronger ability to focus on relevant regions such as instrument tips and active interaction areas. These enhanced attention patterns demonstrate the effectiveness of auxiliary queries in guiding the model toward clinically important features. The aggregated heatmaps from the multi-head attention mechanism further align with areas of surgical interest, such as tool–tissue contact zones, confirming the benefits of our query-based design in refining spatial focus.

**Figure 5:**
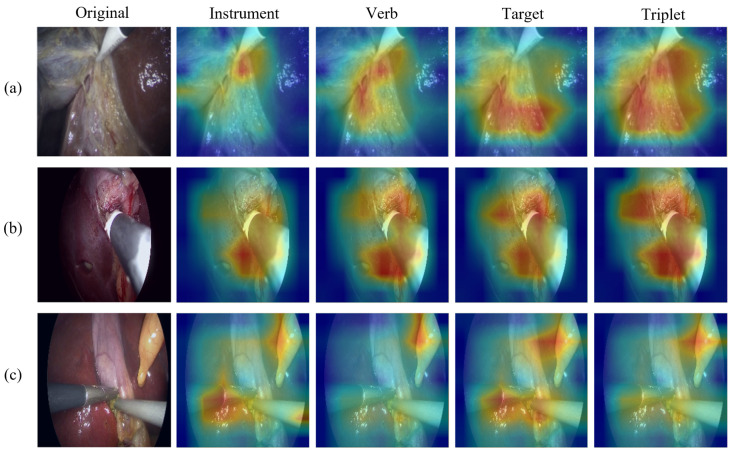
Visualization of attention maps from different task-specific query groups (instrument, verb, target) across three representative frames (rows (**a**–**c**)). The triplet attention map is produced by the decoding head responsible for triplet classification. The visualization demonstrates how each query group focuses on distinct but complementary semantic regions, reflecting their respective recognition objectives and highlighting the task-awareness of our multi-query decoding framework.

Figure 5 presents a comprehensive visualization of attention maps generated by different query groups across three representative surgical frames. Each row corresponds to a representative video frame, with the first column displaying the original image, followed by attention maps generated by three task-specific query decoding heads: instrument, verb, and target. The last column presents the attention map produced by the triplet classification head. The instrument query consistently concentrates on surgical tools such as graspers or hooks; the verb query broadens the focus to include both instruments and dynamic interaction regions; the target query highlights the relevant anatomical structures undergoing manipulation or dissection. These results demonstrate that task-specific queries effectively capture distinct semantic cues, enabling the model to learn disentangled and interpretable representations aligned with the visual semantics of each subtask. Moreover, the results confirm that attention distributions across task-specific queries remain specialized and non-redundant.

These visualizations collectively demonstrate that the proposed multi-query architecture facilitates semantic separation, producing more localized and interpretable attention patterns compared to baseline methods. By guiding the model to focus on task-relevant regions, the auxiliary queries improve both recognition performance and model interpretability for potential clinical applications.

**Figure 6:**
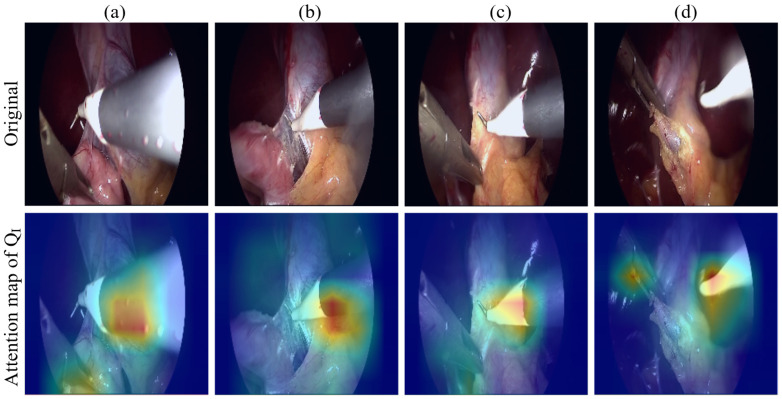
Temporal attention visualization of the instrument query $Q_{I}$ across consecutive frames. Each column (**a**–**d**) represents one frame from a continuous surgical sequence. The top row shows the original frames, and the bottom row overlays the attention maps of $Q_{I}$ generated by the TKQ module. The color gradient highlights the regions where the query focuses in each frame, illustrating how the model maintains temporal coherence and consistently attends to the instrument across time, thereby enhancing the stability and reliability of recognition.

To further analyze the temporal modeling behavior of the proposed TKQ module, we visualize the attention maps of the instrument query $Q_{I}$ over a short sequence of consecutive frames, as shown in Figure 6. This visualization highlights how $Q_{I}$ propagates its focus across time through query updates guided by the previous frame. The attention regions exhibit smooth transitions and consistently emphasize relevant anatomical structures and surgical instruments. In these four consecutive frames, the grabber, typically located on the left, and the hook, typically located on the right, are effectively tracked during movement, with spatial regions receiving continuous attention across frames clearly highlighted. These results demonstrate that the model maintains temporal coherence and that the attention flow of $Q_{I}$ aligns well with the expected continuity of surgical actions, providing qualitative evidence for the temporal interpretability of our method.

### 5.3. Prediction Result Visualization

To further assess the classification performance of our model, we visualize the frame-wise prediction results of the auxiliary tasks on the CholecT45 test set and compare them with the ground-truth annotations and predictions from the Rendezvous baseline [18]. Due to the challenge of directly visualizing the full 100-way triplet classification, we decompose the task into three subtasks, including instrument, verb, and target, and generate color-coded visualizations for each.

**Figure 7:**
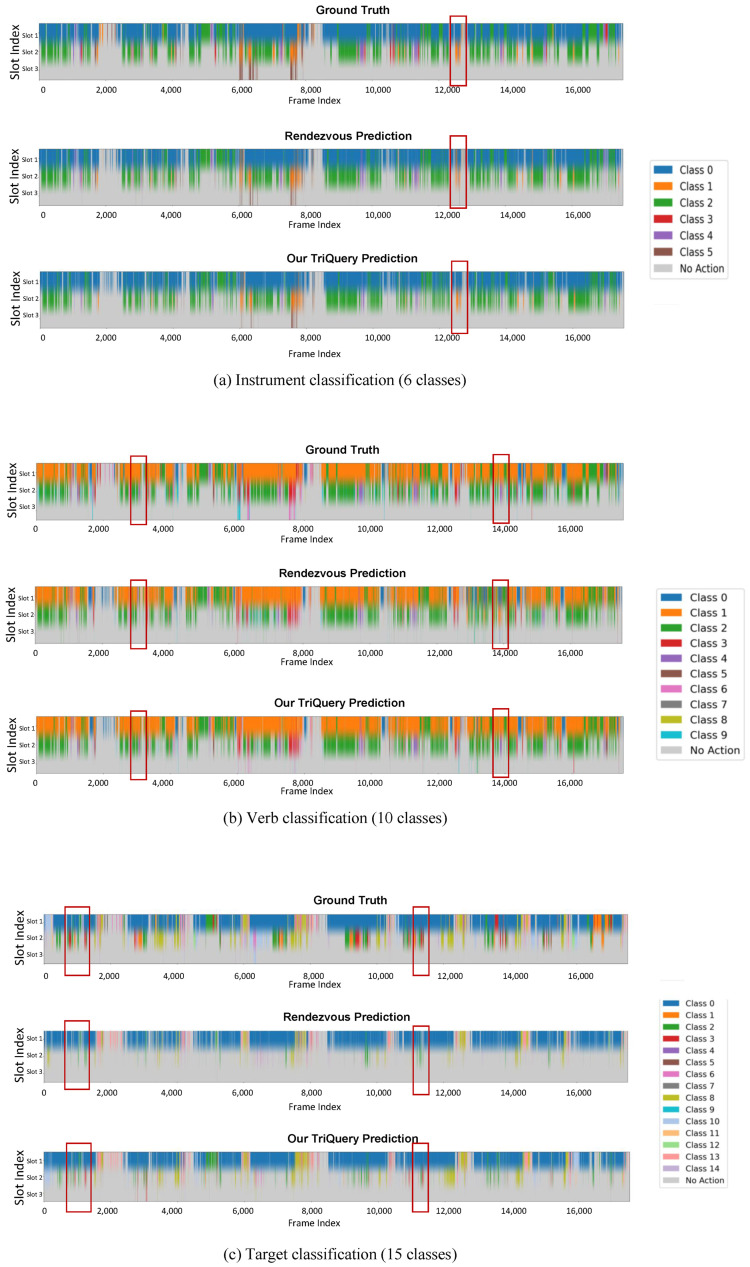
Visualization of classification results on the CholecT45 test set, comparing the ground truth, Rendezvous baseline, and our TriQuery model. (**a**) Instrument classification (6 classes), (**b**) verb classification (10 classes), and (**c**) target classification (15 classes). All nine test videos are concatenated along the horizontal axis, while the vertical axis displays up to three concurrent label slots to accommodate multiple co-occurring labels, such as simultaneous instrument usage. This stacked color representation allows intuitive comparison of prediction accuracy and temporal consistency across models.

Figure 7a–c present visualizations for instrument, verb, and target classification, respectively. Each figure illustrates a timeline of predictions for all frames across the nine test videos (totaling approximately 16,000 frames). The horizontal axis represents the temporal progression of concatenated frames, while the vertical axis contains three label slots per frame to accommodate concurrent actions, such as the simultaneous use of multiple tools. For each frame, the top row displays the ground truth, the middle row shows predictions from the Rendezvous method, and the bottom row presents results from our TriQuery model. Distinct colors are assigned to each class and shown in the legend to facilitate intuitive comparison. The consistent layout across subplots allows direct visual comparison between models and tasks.

The predictions from TriQuery exhibit strong alignment with the ground truth, demonstrating high frame-level accuracy and smooth temporal transitions. Minor discrepancies are primarily observed during visually challenging conditions, such as occlusion by smoke or abrupt camera motion. In contrast, predictions from Rendezvous tend to fluctuate more and show greater temporal noise. Among the three subtasks, the target classification appears more fragmented, which likely reflects higher intra-class variability and greater semantic ambiguity. Nevertheless, TriQuery still maintains notable consistency with the ground truth. Compared to Rendezvous, our model demonstrates improved performance, especially in the red-highlighted regions, where it more accurately captures fine-grained transitions in challenging frames. This observation aligns well with the quantitative improvements reported in Table 1 and Table 2.

These visualizations also offer additional insights into the behavior of our model. The task-specific queries are capable of learning distinct semantic focuses for instrument, verb, and target recognition. Moreover, the auxiliary query branches stabilize the triplet head by providing dense gradients, which are particularly beneficial for underrepresented categories. The TKQ module further enhances inter-frame consistency without relying on computationally expensive components such as optical flow or 3D convolutions, making our approach suitable for surgical decision support and robotic control.

In summary, the visual comparisons across tasks demonstrate the temporal stability and consistency of our model’s predictions while also highlighting specific frame intervals where errors are likely to occur. These findings offer valuable guidance for future improvements and clinical deployment.

## 6. Discussion

### 6.1. Ablation Study: Task Queries and Temporal Guidance

To evaluate the effectiveness of the proposed MQ-DH and TKQ modules, we conducted an ablation study across the instrument, verb, target, and triplet classification tasks. Starting from a baseline model that performs 100-way triplet classification without a query mechanism, we progressively introduced the MQ-DH and TKQ modules into the TriQuery framework. We then assessed both the overall triplet classification performance and the individual accuracy of each subtask (instrument, verb, target). All experiments were conducted using five-fold cross-validation on the CholecT45 dataset. This setup allows us to systematically examine the contribution of each module to the final classification performance and validate the design choices of the proposed architecture.

As shown in Table 3, the baseline model, which uses only the Swin Transformer to extract hierarchical spatiotemporal features, achieved an average triplet classification accuracy of 68.10% and an AP of 40.15%. Upon introducing the MQ-DH module, the model’s performance improved significantly, reaching 71.32% accuracy and 45.18% AP. Further incorporating the TKQ module led to additional gains, demonstrating the benefit of incorporating temporal context via task-specific guidance. When both modules were integrated, the model achieved the highest performance across all metrics, confirming the complementary nature and effectiveness of the proposed design. These results collectively validate the contribution of each component and highlight the importance of both task-disentangled query learning and temporal consistency for improving performance in fine-grained surgical phase classification.

### 6.2. Ablation Study: Module Design Analysis

To further validate the design of the proposed MQ-DH module, we conduct an ablation study to assess the individual and combined contributions of its components. Table 4 presents the results across instrument, verb, target, and triplet classification tasks using multiple evaluation metrics.

The first row reports the baseline performance using the Swin Transformer backbone without any query mechanism. The following rows progressively introduce the instrument query ($Q_{I}$), verb query ($Q_{V}$), and target query ($Q_{T}$) to examine their respective impact. The sixth row includes all three task-specific queries, representing the full MQ-DH design, while the final row adds an additional fused triplet query ($Q_{\mathrm{Tri}}$) for comparison, which, as shown, does not yield further improvement.

The last two columns of Table 4 summarize the average triplet classification performance and the best single-fold result. The former reflects the model’s generalization ability across varying subsets, which is important in the context of severe class imbalance, while the latter highlights its performance potential under favorable data distributions. Since Table 4 focuses on the fine-grained design of the MQ-DH’s internal query composition, we primarily describe the triplet (best) performance in the following analysis to better reflect the upper-bound capability of each configuration. In contrast, the performance results in the last column of Table 1, along with Table 2 and Table 3, are reported as five-fold averages to ensure fair and benchmarking across methods.

Focusing on the triplet classification task (100 classes), we observe that the baseline model achieves an average accuracy of 72.24% and an AP of 43.05%. Adding task-specific queries sequentially results in consistent improvements. When all three queries are incorporated, accuracy rises to 75.55% and AP to 48.63%, representing gains of 3.31% and 5.58% over the baseline, respectively. These results support the effectiveness of decomposing the triplet task into structured subtasks and demonstrate that task-specific queries enhance semantic discrimination while mitigating class imbalance.

Notably, introducing the fused triplet query ($Q_{\mathrm{Tri}}$) slightly reduces performance, confirming the advantage of disentangled task-specific query design over naive query fusion. This also validates our decision to exclude the fused query in the final architecture.

### 6.3. Opportunities for Enhancement

While the proposed TriQuery framework demonstrates strong performance on the CholecT45 dataset, several opportunities remain for further refinement. At present, the TKQ module leverages only the immediately preceding frame to model temporal context. This design supports causal inference and efficient processing, making it suitable for real-time deployment, and is partly motivated by the characteristics of surgical video data, where sudden scene changes or frame drops can occur. However, this short-term focus may limit the ability to capture long-range dependencies in complex procedural workflows. Future extensions could explore incorporating richer temporal context through memory banks, sliding-window attention, or other temporal aggregation strategies. Moreover, while the current fusion weight α in TKQ and the loss weights were empirically chosen to ensure stable convergence and performance, they have been retained as tunable hyperparameters to support future extensions. Incorporating dynamic weighting mechanisms that adapt to different surgical scenarios or training stages may further enhance the model’s robustness and generalizability.

Another promising direction for improvement is to enhance the flexibility and adaptability of the query design. While the current use of predefined task-specific queries aligns well with the dataset’s triplet annotation scheme and offers clear interpretability, it may not generalize effectively to datasets with different task formulations or structures. This limitation motivates the exploration of dynamic query generation strategies, such as those informed by self-supervised learning or hierarchical clustering, which could enable broader adaptability without sacrificing semantic clarity. Furthermore, as the effectiveness of auxiliary queries (instrument, verb, and target) can vary across procedural contexts, incorporating adaptive weighting or dynamic query routing mechanisms may further improve the model’s robustness and overall performance.

In addition, we aim to incorporate domain-specific prior knowledge into query initialization and evaluate the framework on broader surgical datasets and deployment scenarios. These directions are critical for building robust, generalizable, and clinically applicable intelligent video understanding systems.

## 7. Conclusions

In this study, we propose a novel query-based multi-task learning framework for fine-grained surgical video action recognition, TriQuery, which leverages the Swin Transformer backbone and a task-oriented query decoder, and incorporates a query update mechanism to address the inherent challenges of class imbalance and temporal dynamics in laparoscopic surgery scenarios. By decomposing the triplet classification task into three semantically meaningful subtasks—instrument, verb, and target—and introducing learnable queries for each task, the model constructs explicit attention on spatially relevant regions. In addition, a cross-frame initialization strategy enhances temporal continuity and context awareness. This design improves interpretability and enhances recognition performance through auxiliary supervision. Experiments on the CholecT45 dataset demonstrate that TriQuery achieves improved performance compared to baseline methods, particularly in recognizing underrepresented categories. It also generates interpretable attention maps that align well with clinical expectations. The model remains lightweight and scalable, making it well-suited for deployment in clinical environments with constrained computational resources. These characteristics underscore the potential of TriQuery for effective integration into intelligent surgical systems. In addition, its query-driven design allows for flexible adaptation to other medical video analysis tasks, including skill assessment, workflow stage recognition, and intraoperative decision support.

Future work will focus on improving long-range temporal modeling through richer aggregation methods, enhancing the adaptability of query mechanisms via dynamic or self-supervised strategies, and incorporating domain knowledge to boost generalization across broader surgical datasets and deployment environments.

## Figures and Tables

**Table 1:** Comparison with state-of-the-art methods for surgical triplet classification on the CholecT45 dataset using five-fold cross-validation. For clarity, results from the first three folds are reported, along with the overall mean performance. Acc, F1, and AP are reported to evaluate triplet classification performance. The best results are highlighted in bold.

Method	Fold 1			Fold 2			Fold 3			Mean		
	Acc	F1	AP	Acc	F1	AP	Acc	F1	AP	Acc	F1	AP
CNN+RNN [4]	71.86	53.46	41.44	70.52	56.57	42.91	69.73	58.59	44.78	69.59	54.52	41.37
CNN-LSTM [24]	72.52	54.87	42.29	69.02	57.91	43.86	68.96	58.01	43.83	70.54	54.18	41.17
Dual-stream CNN [25]	73.06	54.65	43.28	70.39	56.41	43.06	70.14	56.69	43.16	70.78	53.56	41.35
3D CNN [5]	74.05	50.65	39.22	69.73	50.62	38.32	**72.96**	53.52	41.64	70.97	49.49	38.06
Rendezvous [18]	72.18	51.23	34.93	69.88	52.14	39.13	49.41	50.11	36.87	69.11	50.97	37.98
Swin-T (Baseline) [6]	72.24	54.47	43.05	67.58	55.57	41.21	68.06	56.75	42.08	68.10	53.69	40.15
TriQuery (ours)	**75.55**	**59.19**	**48.63**	**71.79**	**60.77**	**47.33**	71.57	**59.85**	**46.70**	**71.53**	**57.79**	**45.19**

**Table 2:** Comparison with state-of-the-art methods on per-component classification tasks (instrument, verb, and target) using the CholecT45 dataset. All metrics are averaged across five folds. For each task, Acc, F1, and AP are reported. The triplet scores are also included for reference. The best results are highlighted in bold.

Method	Instrument			Verb			Target			Triplet		
	Acc	F1	AP	Acc	F1	AP	Acc	F1	AP	Acc	F1	AP
CNN+RNN [4]	95.99	81.00	85.80	94.81	71.47	69.55	84.67	63.00	54.89	69.59	54.52	41.37
CNN-LSTM [24]	96.04	81.04	85.95	94.79	71.48	69.38	84.09	62.81	54.60	70.54	54.18	41.17
Dual-stream CNN [25]	95.99	80.98	85.76	94.82	71.39	69.73	84.78	62.73	54.67	70.78	53.56	41.35
3D CNN [5]	95.96	80.97	85.64	94.69	70.29	68.77	**84.93**	60.75	53.09	70.97	49.49	38.06
Rendezvous [18]	95.48	79.23	84.08	94.51	69.28	67.67	82.65	53.22	46.33	69.11	50.97	37.98
Swin-T (Baseline) [6]	95.91	81.14	85.41	94.70	71.53	69.05	83.59	62.39	53.54	68.10	53.69	40.15
TriQuery (ours)	**96.88**	**83.51**	**88.67**	**98.29**	**74.30**	**71.82**	84.00	**66.47**	**57.69**	**71.53**	**57.79**	**45.19**

**Table 3:** Ablation study of the Multi-Query Decoding Head (MQ-DH) and the Top-K Guided Query Update (TKQ) module on the CholecT45 dataset. ✓ indicates that the corresponding component is enabled. The comparison demonstrates the individual and combined effectiveness of MQ-DH and TKQ. The best results are highlighted in bold.

MQ-DH	TKQ	Instrument			Verb			Target			Triplet		
		Acc	F1	AP	Acc	F1	AP	Acc	F1	AP	Acc	F1	AP
		95.91	81.14	85.41	94.70	71.53	69.05	83.59	62.39	53.54	68.10	53.69	40.15
✓		**96.90**	83.50	**88.82**	95.28	74.29	**72.29**	83.91	**66.48**	57.60	71.32	57.78	45.18
✓	✓	96.88	**83.51**	88.67	**98.29**	**74.30**	71.82	**84.00**	66.47	**57.69**	**71.53**	**57.79**	**45.19**

**Table 4:** Ablation study on the query composition of the MQ-DH on the CholecT45 dataset. We progressively introduce instrument ($Q_{I}$), verb ($Q_{V}$), and target ($Q_{T}$) task-specific queries. The last row additionally includes a fused triplet query ($Q_{\mathrm{Tri}}$) for comparison, which does not yield further improvement and is not included in the final architecture. The final column (Triplet (Best)) reports the best fold performance across five-fold cross-validation. The best results are highlighted in bold.

$Q_{I}$	$Q_{V}$	$Q_{T}$	$Q_{\mathrm{Tri}}$	Instrument			Verb			Target			Triplet			Triplet (Best)		
				Acc	F1	AP	Acc	F1	AP	Acc	F1	AP	Acc	F1	AP	Acc	F1	AP
				95.91	81.14	85.41	94.70	71.53	69.05	83.59	62.39	53.54	68.10	53.69	40.15	72.24	54.47	43.05
✓				**96.89**	83.41	**88.83**	–	–	–	–	–	–	70.88	57.73	45.07	73.52	58.84	47.21
	✓			–	–	–	95.23	74.08	72.03	–	–	–	71.04	57.15	45.08	74.21	58.13	48.33
		✓		–	–	–	–	–	–	83.81	66.26	57.39	71.07	57.52	44.97	74.47	58.77	47.77
✓	✓			96.91	83.47	88.78	95.28	74.21	**72.28**	–	–	–	71.49	57.79	45.12	75.43	58.49	48.04
✓	✓	✓		96.88	**83.51**	88.67	**98.29**	**74.30**	71.82	**84.00**	**66.47**	**57.69**	**71.53**	**57.79**	**45.19**	**75.55**	**59.19**	**48.63**
✓	✓	✓	✓	96.79	83.32	88.35	95.18	73.92	71.74	83.75	66.06	56.83	70.55	57.77	44.84	73.92	59.18	47.40

## Data Availability

The data presented in this study were composed of the dataset CholecT45. The CholecT45 dataset is available at https://github.com/CAMMA-public/cholect45 (accessed on 21 August 2025).

## References

[1] Bain A.P., Holcomb C.N., Zeh III H.J., Sankaranarayanan G. (2024). Artificial intelligence for improving intraoperative surgical care. Glob. Surg. Educ.-J. Assoc. Surg. Educ..

[2] Lukács E., Levendovics R., Haidegger T. (2023). Enhancing autonomous skill assessment of robot-assisted minimally invasive surgery: A comprehensive analysis of global and gesture-level techniques applied on the JIGSAWS dataset. Acta Polytech. Hung.

[3] Aspart F., Bolmgren J.L., Lavanchy J.L., Beldi G., Woods M.S., Padoy N., Hosgor E. (2022). ClipAssistNet: Bringing real-time safety feedback to operating rooms. Int. J. Comput. Assist. Radiol. Surg..

[4] Islam M.M., Islam M.Z., Asraf A., Al-Rakhami M.S., Ding W., Sodhro A.H. (2022). Diagnosis of COVID-19 from X-rays using combined CNN-RNN architecture with transfer learning. Benchcouncil Trans. Benchmarks Stand. Eval..

[5] Liu H., Tu J., Liu M. (2017). Two-stream 3d convolutional neural network for skeleton-based action recognition. arXiv.

[6] Liu Z., Ning J., Cao Y., Wei Y., Zhang Z., Lin S., Hu H. Video swin transformer. Proceedings of the IEEE/CVF Conference on Computer Vision and Pattern Recognition.

[7] Bao F., Nie S., Xue K., Cao Y., Li C., Su H., Zhu J. All are worth words: A vit backbone for diffusion models. Proceedings of the IEEE/CVF Conference on Computer Vision and Pattern Recognition.

[8] Jiao E., Leng Q., Guo J., Meng X., Wang C. (2025). Vision Transformer with window sequence merging mechanism for image classification. Appl. Soft Comput..

[9] Luvizon D.C., Picard D., Tabia H. (2020). Multi-task deep learning for real-time 3D human pose estimation and action recognition. IEEE Trans. Pattern Anal. Mach. Intell..

[10] Gan Z., Jin L., Nie L., Wang Z., Zhou L., Li L., Wang Z., Li J., Xing J., Zhao J. ASQuery: A query-based model for action segmentation. Proceedings of the 2024 IEEE International Conference on Multimedia and Expo (ICME).

[11] Zhang Y., Yang Q. (2018). An overview of multi-task learning. Natl. Sci. Rev..

[12] Jia Y., Dang R., Wang D., Wu Z., Yang T., Tian Z., Yin J. Visual SLAM for Dynamic Environment Using Pre-Frame Semantic. Proceedings of the 2024 IEEE International Conference on Unmanned Systems (ICUS).

[13] Wang L., Zang J., Zhang Q., Niu Z., Hua G., Zheng N. (2018). Action recognition by an attention-aware temporal weighted convolutional neural network. Sensors.

[14] Kim H.W., Choi Y.S. (2024). Fusion attention for action recognition: Integrating sparse-dense and global attention for video action recognition. Sensors.

[15] Wei W., Zhu C., Hu L., Liu P. (2025). Application of a Transfer Learning Model Combining CNN and Self-Attention Mechanism in Wireless Signal Recognition. Sensors.

[16] Xu Y., Wang Y. (2025). Single-Image Super-Resolution via Cascaded Non-Local Mean Network and Dual-Path Multi-Branch Fusion. Sensors.

[17] Carion N., Massa F., Synnaeve G., Usunier N., Kirillov A., Zagoruyko S. (2020). End-to-end object detection with transformers. Computer Vision—ECCV 2020, Proceedings of the 16th European Conference on Computer Vision, Glasgow, UK, 23–28 August 2020.

[18] Nwoye C.I., Yu T., Gonzalez C., Seeliger B., Mascagni P., Mutter D., Marescaux J., Padoy N. (2022). Rendezvous: Attention mechanisms for the recognition of surgical action triplets in endoscopic videos. Med. Image Anal..

[19] Tufail H., Naseer A., Tamoor M., Ali A.R. (2024). Advancements in Query-Based Tabular Data Retrieval: Detecting Image Data Tables and Extracting Text using Convolutional Neural Networks. Preprint.

[20] Liu C., Zhang B., Bo C., Wang D. (2024). Query-Based Object Visual Tracking with Parallel Sequence Generation. Sensors.

[21] Zhou S., Yang P., Wang J., Luo Y., Loy C.C. Upscale-a-video: Temporal-consistent diffusion model for real-world video super-resolution. Proceedings of the IEEE/CVF Conference on Computer Vision and Pattern Recognition.

[22] Hori S., Omi K., Tamaki T. (2024). Query matching for spatio-temporal action detection with query-based object detector. arXiv.

[23] Nwoye C.I., Padoy N. (2022). Data splits and metrics for benchmarking methods on surgical action triplet datasets. arXiv.

[24] Wang Z., Song Y., Pang L., Li S., Sun G. (2025). Attention-Enhanced CNN-LSTM Model for Exercise Oxygen Consumption Prediction with Multi-Source Temporal Features. Sensors.

[25] Gong S., Yan X., Fang Y., Paul A., Wu Z., Chen J. (2024). A dual-stream CNN-BiLSTM for human motion recognition with raw radar data. IEEE Sens. J..

